# Detection of *Mycobacterium tuberculosis* by Using Loop-Mediated Isothermal Amplification Combined with a Lateral Flow Dipstick in Clinical Samples

**DOI:** 10.1155/2013/926230

**Published:** 2013-03-05

**Authors:** Thongchai Kaewphinit, Narong Arunrut, Wansika Kiatpathomchai, Somchai Santiwatanakul, Pornpun Jaratsing, Kosum Chansiri

**Affiliations:** ^1^Innovative Learning Center, Srinakharinwirot University, Sukhumvit 23, Bangkok 10110, Thailand; ^2^Centex Shrimp, Faculty of Science, Mahidol University, Bangkok 10400, Thailand; ^3^National Center for Genetic Engineering and Biotechnology (BIOTEC), National Science and Technology Development Agency, Pathumthani 12120, Thailand; ^4^Department of Pathology, Faculty of Medicine, Srinakharinwirot University, Sukhumvit 23, Bangkok 10110, Thailand; ^5^Department of Biochemistry, Faculty of Medicine, Srinakharinwirot University, Sukhumvit 23, Bangkok 10110, Thailand

## Abstract

Tuberculosis (TB) is a communicable disease caused by the bacterium *Mycobacterium tuberculosis* (MTB) and is a persistent problem in the developing countries. Loop-mediated isothermal amplification (LAMP) allows DNA to be amplified rapidly at a constant temperature. Here, a LAMP method was combined with a chromatographic lateral-flow dipstick (LFD) to detect IS*6110* gene of *M. tuberculosis* specifically and rapidly. The reaction was optimized at 63°C for 60 min, and the amplified DNA hybridized to an FITC-labeled oligonucleotide probe for 5 min was detected at the LFD test line 5 min after application. Excluding the step of DNA extraction, the test results could be generated approximately within 1 h. In addition to the advantage of short assay time, this technique could avoid the contact of carcinogenic ethidium bromide due to the exclusion of the electrophoresis analysis step. Furthermore, the data indicated that LAMP-LFD could detect *M. tuberculosis* genomic DNA as little as 5 pg. The technique showed a significant specificity since no cross-hybridization to *M. intracellulare* (MIC), *M. fortuitum* (MFT), *M. avium* (MAV), *M. kansasii* (MKS), and *M. gordonae* (MGD) genomic DNAs was observed. In the clinical unknown samples test, the sensitivity of LAMP-LFD was 98.92** **% and the specificity was 100** **% compared to those of the standard culture assay. Based on its sensitivity, specificity, rapidity, low cost, and convenience, LAMP-LFD could be applicable for use in both laboratories and epidemiological surveys of MTB.

## 1. Introduction

 TB is an airborne disease caused by the bacterium *Mycobacterium tuberculosis* (MTB). WHO reported that TB is a persistent problem in developing countries and ranks as the second leading cause of death from an infectious disease worldwide after the human immunodeficiency virus (HIV) [1]. This bacterium is a slow-growing bacterium that needs 1-2 months for growing in a culture; however, a rapid and timely diagnosis of tuberculosis is essential to combat this disease. The Ziehl-Neelsen (ZN) stain for direct specimen examination is a conventional diagnostic tool but lacks sensitivity. The tests based on PCR have shown promise for the detection of mycobacteria in clinical samples [2–4], but this amplification process requires additional processing time, reagents, and devices, which affect the cost of the assay. Moreover, PCR analysis needs well-trained personnel.

The LAMP assay allows DNA to be amplified at a constant temperature of 60–65°C [5]. After LAMP, the amplified DNA is normally detected by agarose gel electrophoresis, ethidium bromide staining, and UV transillumination. Due to the use of several primers, LAMP generates a complex mixture of DNA products of different sizes; therefore, gel analysis cannot distinguish specific and nonspecific products. To avoid possible false positive results, the authenticity of LAMP DNA products can be confirmed by restriction endonuclease digestion [5] or by hybridization to specific probes [6]. Later, to further simplify and shorten the time needed to generate LAMP data, a biotin-labeled oligonucleotide probe and an FITC-labeled DNA probe captured by gold-labeled anti-FITC antibody following chromatography on a LFD (Milenia GenLine Hybri Detect) were developed. The techniques were successfully applied in the detection of viruses such as shrimp infectious hypodermal and hematopoietic necrosis virus [7] and shrimp Taura syndrome virus [8]. Using this strategy, the sensitivity is equivalent to that of PCR assay with similar time use of approximately 1 h. Described here was a LAMP-LFD method optimized for the detection of MTB. The sensitivity and specificity of this technique were investigated in comparison to those of a PCR assay.

## 2. Materials and Methods 

### 2.1. Samples and DNA Extraction

 All clinical samples (101 unknown sputum samples) and standard strain (H37RVKK11-20) were provided from the National Tuberculosis Reference Laboratory (NTRL), Bureau of Tuberculosis, Department of Disease Control, Ministry of Public Health, Thailand. DNA was extracted from all clinical samples following the method of Rienthong et al. [9]. Briefly, Sputum unknown samples were decontaminated with N-acetyl-L-cysteine-sodium hydroxide. After centrifugation, the crude cell lysates were suspended in 300 *μ*L of distilled water, killed with heat at 95°C for 20 min in Thermoblock, and then underwent sonification for 15 min at the highest speed in an ultrasonic bath, followed by spinning the samples in a standard centrifuge with an aerosol-tight rotor at approximately 10,000 g for 5 min. The supernatant was used for the LAMP amplification. The total samples of unknown sputum were compared to those of the standard culture assay on Lowenstein-Jensen slants and incubated at 37 °C for 8 weeks. The genomic DNA from the standard (H37RVKK11-20) was prepared following the method of Kaewphinit and coworkers [10]. The concentration and quality of genomic DNA in the LAMP reaction were determined by spectrophotometric analysis at 260 and 280 nm. 

### 2.2. LAMP Primers and PCR Primers

**Figure 1:**
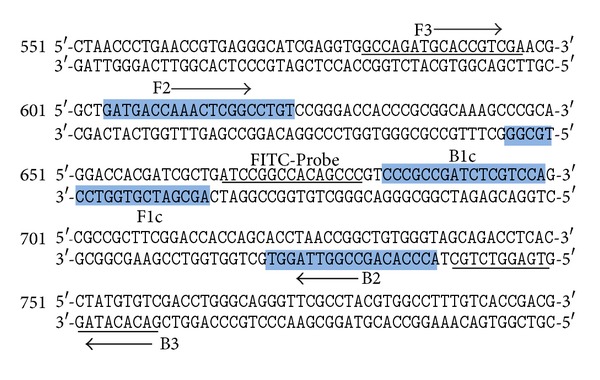
Nucleotide sequence of IS*6110* gene (GenBank accession number: X17348). The primers F3 and B3 were shown as underlined nucleotide sequences and arrows. The FIP (F1c/TTTT/F2) and BIP (B1c/TTTT/B2) inner primers are the shaded boxes and arrows. The FITC-labeled probe sequence is shown in italic and underlined sequences.

 LAMP primers for MTB were designed according to the published sequences of the IS*6110* gene specific for MTB genome (Gen-Bank accession no. X17348) using Primer Explorer version 4 (http://primerexplorer.jp/elamp4.0.0/index.html). The directions and details of the primers are shown in Figure 1 and Table 1. The normal primers and biotin-labeled FIP primer were synthesized by Bio Basic Inc., Canada.

### 2.3. Optimization of Temperature for LAMP

To determine the optimum temperature for amplification, the LAMP reactions were carried out at 60, 63, and 65°C for 1 h, followed by the analysis of the LAMP products by gel electrophoresis. The reaction mixture contained 2 *μ*M, each of inner primers FIP and BIP, 0.2 *μ*M, each of outer primers F3 and B3, 1.6 mM of dNTP mix (Promega, Madison, WI, USA), 0.5 M of betaine (Sigma-Aldrich, St. Louis, MO, USA), 6 mM of MgSO_4_, 8U of Bst DNA polymerase (large fragment; New England Biolabs Inc., Beverly, MA, USA), 1X of the supplied buffer, and 50 ng of DNA in a final volume of 25 *μ*L.

### 2.4. Lateral Flow Dipstick (LFD) Assay

 A 5′-FITC-labeled oligonucleotide probe designed according to the IS*6110* gene of MTB between the F1c and B1c primer targets was synthesized by Bio Basic Inc., Canada. As recommended in similar tests [7, 8], 20 picomoles of FITC-labeled probe (FITC-5′-ATCCGGCCACAGCCC-3′) was added to the LAMP reaction and after hybridization at 65°C for 5 min, 8 *μ*L hybridized product was added to 120 *μ*L assay buffer in a new tube, and an LFD was dipped into the mixture for 5 min.

### 2.5. Sensitivity of LAMP by Gel Electrophoresis and LFD

To determine detection sensitivity limits, 10-fold serial dilutions (10^−1^ to 10^−6^) of 50 ng of total DNA of MTB (H37RVKK11-20) were used as templates for biotin-labeling LAMP tests performed under optimized conditions. Amplified DNA was detected either by 2% agarose gel electrophoresis and ethidium bromide staining followed by DNA visualization using a UV transilluminator or by the use of LFD as described above.

### 2.6. Specificity of LAMP-LFD

The specificity of LAMP primers was examined using 50 ng of total DNA extracted from other mycobacterium. These included infectious *M. intracellulare* (MIC), *M. fortuitum* (MFT), *M. avium* (MAV), *M. kansasii* (MKS), and *M. gordonae* (MGD). The biotin-labeled LAMP products were analyzed by 2% agarose gel electrophoresis and by LFD.

### 2.7. PCR for MTB Detection

Tenfold serial dilutions of 50 ng of total DNA extracted from MTB (H37RVKK11-20) were used as the template for PCR detection of MTB using the Thermal Cycler (Touchgene Gradient, model: FTGRAD2D, Techne Ltd, UK). All reactions were 25 *μ*L volume containing 50 ng of genomic DNA in 10x PCR buffer, 1 *μ*M, each of primers, 100 *μ*M of dNTP, 1.5 mM MgCl_2_, and 1.5 units of *Taq* DNA polymerase (Invitrogen). PCR was performed by using a DNA thermal cycler for 30 cycles. Each cycle consisted of denaturation at 94°C for 2 min, annealing at 53°C for 1 min, and extension at 72°C for 1 min and a final extension at 72°C for 10 min. PCR amplicon was analyzed by 1.5% agarose gel electrophoresis. The PCR assay was prepared following the method of Kaewphinit and colleagues [10]. 

### 2.8. Bacterial DNA Samples Analysis

 The LAMP-LFD assays in this study for identification of 101 clinical sputum double-blind samples were detected individually using the LFD assay compared to those of the standard culture assay and AFB smear.

## 3. Results 

### 3.1. Optimization of Reaction Temperature for MTB Detection

**Figure 2:**
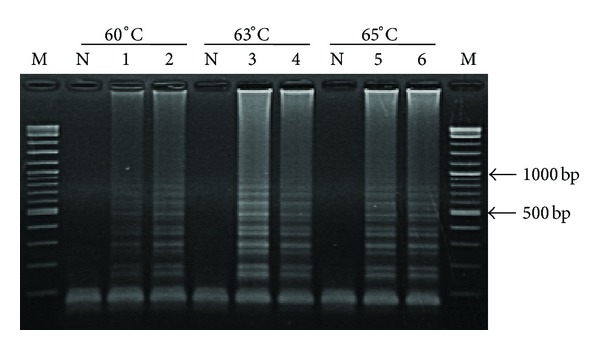
Optimization of biotin labeling LAMP conditions at different temperatures (60, 63, and 65°C) by using 50 ng (lanes 1, 3, and 5) and 5 ng (lanes 2, 4, and 6) of MTB genomic DNA. Lanes M and N represent DNA ladder marker and negative control (no-DNA template), respectively.

When the LAMP was carried out at 60, 63, and 65°C for 60 min with 50 ng of MTB infected samples DNAs (Figure 2), all three temperatures tested generated similar LAMP product patterns with the clearest and strongest bands at 63°C. Based on this, the temperature of 63°C was used for all tests in this study.

### 3.2. Comparison of LAMP and PCR Sensitivity with Gel Electrophoresis

**Figure 3:**
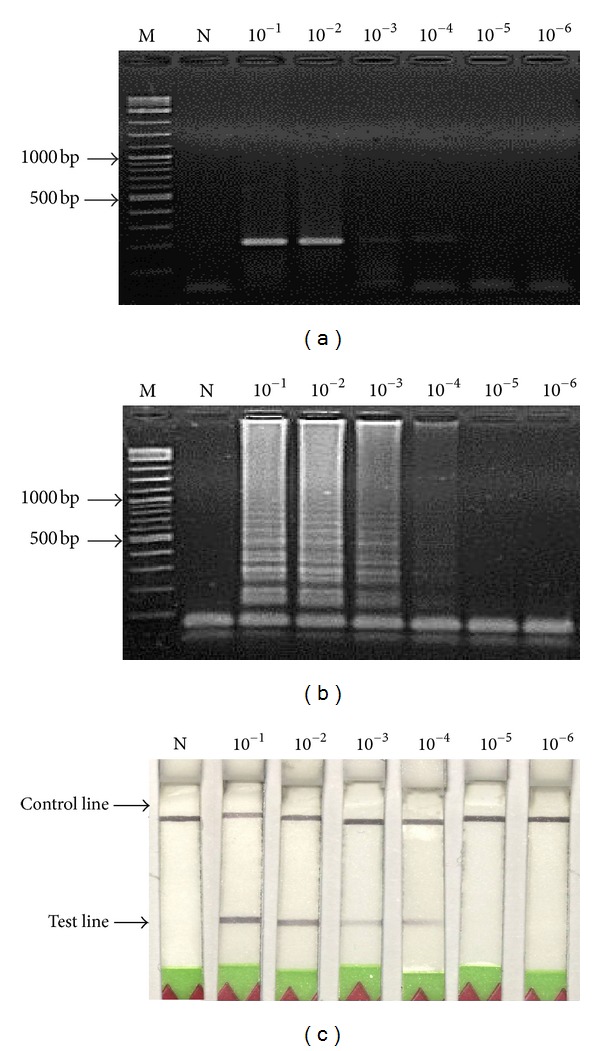
Detection sensitivity data of MTB genomic DNAs at concentration range of 10^−1^ to 10^−6^ dilutions (initial concentration was 50 ng) obtained from (a) PCR, (b) LAMP, and (c) LAMP-LFD. Lanes M and N represent DNA ladder marker and negative control (no-DNA template), respectively.

Using equivalent quantities of DNA extracted from MTB-infected samples as templates at various dilutions, the detection limits for LAMP and PCR were both at 10^−4^ dilution (Figures 3(a) and 3(b)). 

### 3.3. Sensitivity of the Combined LAMP-LFD

The LAMP-LFD detection of MTB showed a limit at 10^−4^ DNA dilution (Figure 3(c)). This corresponded to the detection limit for LAMP or PCR methods followed by electrophoresis, as described above. The detection limit was also similar to those previously reported for hepatopancreatic parvovirus (HPV) detection in black tiger shrimp *Penaeus monodon *[11]. 

### 3.4. Specificity of LAMP-Gel Electrophoresis and LAMP-LFD

**Figure 4:**
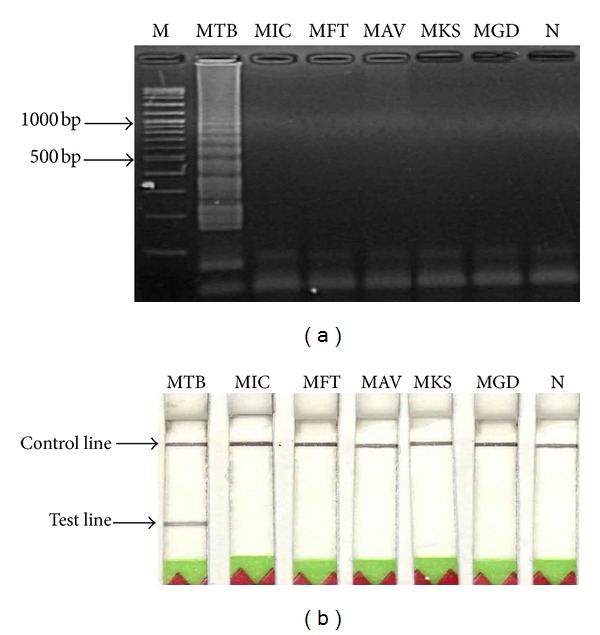
Specificity test data of the LAMP method for MTB using 50 ng each of DNA templates and detection by (a) gel electrophoresis or by (b) LFD. Lane M represents DNA ladder marker. Lanes 1–6 represent DNAs of MTB, MIC, MFT, MAV, MKS, and MGD, respectively. Lane 7 represents negative control (no-DNA template).

A specificity test was manipulated by using 50 ng each of MTB genomic DNAs and other mycobacteria (i.e., MIC, MFT, MAV, MKS, and MGD). The data revealed that no cross-reactions were obtained from LAMP-gel electrophoresis (Figure 4(a)) and LAMP-LFD or LFD (Figure 4(b)).

### 3.5. Bacterial DNA Samples Analysis

 A total of 101 clinical sputum double-blind samples using culture and AFB smear test results were confirmed by the NTRL. After LAMP-amplification, all genomic DNAs were hybridized with a FITC-Probe, and the results were detected by LFD and compared with those obtained from the traditional culture and AFB smear test. In brief, the sensitivity and specificity of this assay for clinical samples' diagnosis of MTB genomic DNAs were 98.92% and 100%, respectively, but the sensitivity of AFB smears test was 80.65%, both methods compared to those of the culture assay (Table 2).

## 4. Discussion

The LAMP-LFD technique was used for the detection of MTB in clinical double blind samples. In this study, the LAMP method was carried out at 63°C for 60 min, which was faster than typical PCR methods that require 2-3 h for PCR cycling. Referring to the test, no expensive equipment was required and the results could be determined within approximately 1 h (not including DNA preparation time). LAMP-LFD was faster than LAMP-gel electrophoresis (2 h 30 min) and much faster than PCR-gel electrophoresis (3 h 30 min) [12].

The LAMP detection method that targeted a 178 bp sequence of the IS*6110* gene was successfully developed for the detection of MTB, with the sensitivity of the LAMP being comparable to that of common PCR amplification for MTB, which was at the concentration level 5 pg of genomic DNA, a sensitivity higher than that reported previously [13]. 

In the clinical unknown sample test (Table 2), the sensitivity of the LAMP-LFD was 98.92* *% (92/93), but AFB smear test was 80.65% (75/93), as compared to those of the culture assay, and the specificity of the LAMP-LFD was 100* *%. For the one sample containing LFD assay was false negative MTB as compared with those of the culture assay. It was reasoned that possibly only a small amount of bacterial DNA was extracted from sputum samples. When compared to positive cultures, the sensitivity, specificity, positive predictive value, and negative predictive value of the method for MTB diagnosis were higher than that method, and the overall time for the procedure was faster than that reported previously [13, 14]. In addition, the LAMP-LFD assay for detection step confirms the identity of the specific amplicon by hybridization and avoids the use of carcinogens such as ethidium bromide. The test platform can be adapted easily for rapid detection of other mycobacteria agents simply by designing appropriate sets of LAMP primers and specific probes. Since the cost for the LAMP-LFD detection is comparable with that for standard PCR followed by electrophoresis, it constitutes a highly sensitive, safe and rapid alternative for the detection of MTB. Hence, the technique could be applicable for detecting minute amounts of MTB in clinical specimens. According to the specificity test, no cross-reactions to other mycobacteria were observed. This indicated that the LAMP method was specific for MTB. The introduction of LFD for analysis of LAMP amplification products could reduce the time and complications associated with usual detection by electrophoresis. This resulted in a total analysis time (excluding the DNA extraction step) of less than 75 min. The high sensitivity and specificity, the relatively short analysis time, and the use of relatively inexpensive equipment were key advantages of the LAMP-LFD assay. In addition, the technique did not involve the use of carcinogens such as ethidium bromide. The test platform can be adapted easily for rapid detection of other mycobacteria agents simply by designing appropriate sets of LAMP primers and specific FITC probes to be used with the generic LFD employed. According to the sensitivity, specificity, less time consumption, low cost and convenience, LAMP-LFD could be applicable for use in both laboratories and epidemiological surveys of MTB.

## Figures and Tables

**Table 1:** Primers and probe used for LAMP of the IS*6110* gene of MTB.

Primer name	Genome position	Sequences 5′-3′
F3	581-597	GCCAGATGCACCGTCGA
B3	759-740	GACACATAGGTGAGGTCTGC
FIP (F1c/TTTT/F2)	664-646/TTTT/604-623	AGCGATCGTGGTCCTG CGG-TTTT-
		GATGACCAAACTCGGCCTGT
BIP (B1c/TTTT/B2)	682-699/TTTT/738-721	TCCCGCCGATCTCGTCCA-TTTTT-
		ACCCACAGCCGGTTAGGT
FITC-Probe	665-680	ATCCGGCCACAGCCC

**Table 2:** Clinical double blind samples identified with LAMP-LFD, tested with specific carrying the IS*6110 *gene probes for genotyping, and compared with the culture and AFB smear test.

Culture test*	AFB smear test*				LAMP-LFD test	
	AFB grade					
	3+	2+	1+	—		
	(30)	(30)	(15)	(26)	Positive	Negative
		Positive		Negative		
Positive (93)		75		18	92	1
Negative (8)		0		8	0	8

Total (101)		75		26	92	9

*Culture and AFB smear test results were confirmed by the NTRL, Bureau of Tuberculosis, Department of Disease Control, Ministry of Public Health, Thailand.
